# Protein nanoparticle-based vaccine candidate produced in *Nicotiana benthamiana* against non-typhoidal *Salmonella enterica* in poultry

**DOI:** 10.3389/fpls.2026.1761000

**Published:** 2026-03-06

**Authors:** Shabnam Shamriz, Philip H. W. Mak, Carly A. Charron, Xianhua Yin, Christopher P. Garnham, Elijah G. Kiarie, Moussa S. Diarra, Rima Menassa

**Affiliations:** 1 London Research and Development Centre, Agriculture and Agri-Food Canada (AAFC), London, ON, Canada; 2 Department of Biology, University of Western Ontario, London, ON, Canada; 3 Guelph Research and Development Centre, AAFC, Guelph, ON, Canada; 4 Department of Animal Biosciences, University of Guelph, Guelph, ON, Canada; 5 Department of Biochemistry, University of Western Ontario, London, ON, Canada

**Keywords:** Brucella sp. lumazine synthase, fusion subunit vaccine, plant-produced nanoparticle-based vaccine, poultry, Salmonella enterica serovars

## Abstract

Non-typhoidal *Salmonella enterica* serovars are enteric pathogens in humans that can be acquired from poultry products. *Salmonella* colonisation in poultry is an important cause of economic losses. Due to challenges in controlling *Salmonella* in poultry and the emergence of well-adapted, antibiotic-resistant serovars, there is a need for innovative control strategies, such as vaccines. In this work, plants were used to produce conserved antigenic epitopes of *Salmonella* FepA, an outer membrane protein involved in iron uptake, genetically fused in tandem to self-assembling lumazine synthase from *Brucella* spp. (BLS). The recombinant proteins were purified, characterized, and their immunogenicity was assessed in chickens. Results indicated that the recombinant proteins assemble into decameric particles. These proteins elicit antigen-specific antibodies in chickens that bind to the *Salmonella’s* cell surface. These results demonstrate that the candidate vaccine has the potential to control *Salmonella* colonization in poultry, helping prevent food chain contamination.

## Introduction

(1)Non-typhoidal *Salmonella* serovars (*Salmonella*) have consistently posed a significant challenge for the poultry industry, causing health risks to both birds and consumers, resulting in significant economic losses (Antunes et al., 2016; Mouttotou et al., 2017). *Salmonella* infection in poultry is problematic because beyond the first days of life, chickens are mostly asymptomatic carriers. However, *Salmonella* infection can impair nutrient absorption and digestion, leading to reduced body weight and feed utilization efficiency in infected birds (Chang et al., 2020). In addition, the inflammatory response triggered by *Salmonella* infection can increase metabolic rate and energy expenditure, further contributing to reduced weight gain and decreased feed efficiency (Kogut et al., 2018). *Salmonella* can also cause liver damage, which can further exacerbate metabolic disturbances in infected chickens (Ramtahal et al., 2022). In response to this ongoing challenge, innovative approaches are emerging to develop preventative strategies that aim to improve safeguarding poultry populations and food safety (Wessels et al., 2021). Vaccination has historically been a powerful tool in disease control, and its potential is magnified when combined with modern biotechnological advancements (Van Immerseel et al., 2005; Desin et al., 2013).

Subunit *Salmonella* vaccines that target surface proteins essential for bacterial viability may elicit stronger immune responses than those targeting internal antigens. The presence of live and intact *Salmonella* within infected tissues underscores the importance of immune recognition of surface proteins for achieving efficient protection (Hansson et al., 2000; Bobbala and Hook, 2016). Iron, essential for life, plays a vital role in host-pathogen interactions. Pathogens have developed advanced mechanisms, including siderophores, to acquire iron (Lan et al., 2025; Schalk, 2025). TonB-dependent outer membrane transporters (TBDTs) are surface-associated proteins involved in iron acquisition in pathogenic bacteria, making them potential targets for vaccine development (Moeck and Coulton, 1998; Klebba et al., 2021). These TBDTs play a crucial role in facilitating the uptake of iron-loaded siderophores across the outer membrane of Gram-negative bacteria (Sheldon et al., 2016). In *Salmonella*, specific TBDTs, including FepA (Ferric Enterobactin receptor), are involved in transporting different types of siderophores-iron (III), highlighting their importance in bacterial survival and suggesting their potential as vaccine targets (Wilson et al., 2016).

Subunit vaccines in the form of soluble antigens often lack effectiveness, therefore strategies involving appropriate adjuvants and optimized formulations aim to enhance their immunogenicity. Alternatively, self-assembling protein nanoparticles such as ferritin (Rodrigues et al., 2021), encapsulin (Lagoutte et al., 2018)and lumazine synthase from various organisms (Zhang et al., 2001; Zylberman et al., 2004) have gained attention for their ability to efficiently enhance immune responses and deliver antigens (Donaldson et al., 2018; Ladenstein and Morgunova, 2020). The repetitive presentation of antigens can trigger a stronger immune response than a single presentation due to the increased likelihood of multiple B-cell receptors binding to the antigens simultaneously (Ladenstein and Morgunova, 2020).

*Brucella* sp. lumazine synthase (BLS), an enzyme naturally involved in the biosynthesis of riboflavin, has been shown to have potential as an antigen-display platform due to its ability to self-assemble into decameric nanoparticles that can display antigens on their surface in an ordered manner (Cristófalo et al., 2025). Furthermore, studies using BLS-based chimeric vaccines demonstrated that the highly ordered decameric structure efficiently cross-links B cell receptors, resulting in robust early B cell activation and downstream germinal center (GC) responses (Berguer et al., 2006). In murine models, BLS-fused antigens induced significantly larger and more persistent GC reactions compared to soluble antigens, accompanied by increased frequencies of antigen-specific GC B cells and T follicular helper cells (Berguer et al., 2012; Alvarez et al., 2013; Rossi et al., 2015). The efficacy of several BLS-based vaccines has been demonstrated, highlighting its adjuvant properties and potent immunogenicity when administered orally or systemically (Berguer et al., 2012; Rossi et al., 2015). This makes BLS an attractive scaffold for subunit vaccine design (Ladenstein and Morgunova, 2020).

Plants have gained recognition as versatile hosts to produce health-enhancing proteins. Plants offer advantages such as scalability, cost-effectiveness, and safety, making them an attractive alternative to traditional production methods. While plant-based protein expression technology holds promise, pharmaceutical companies often hesitate to invest significant resources in gaining regulatory approval for new platforms when established expression systems already have regulatory clearance (Schillberg et al., 2019). In the realm of animal vaccines, progress is often faster since there are fewer regulatory hurdles to overcome. Therefore, the use of plants for animal vaccines holds appeal due to their possible rapid development and approval (Takeyama et al., 2015). In addition, plants are consumed by animals as part of their regular diet. Thus, plants offer the possibility to produce oral therapeutic products/vaccines for animals without the need for expensive purification.

In this study, a fusion protein that combines predicted extracellular antigenic peptides from *Salmonella* FepA with BLS was created. This protein was produced in *Nicotiana benthamiana* and its immunogenicity was evaluated in chicks. Elevated levels of IgY following immunization suggests that this nanoparticle vaccine candidate may have potential for further development to prevent *Salmonella* colonization in poultry.

## Materials and methods

### Vaccine construct design

**Figure 1:**
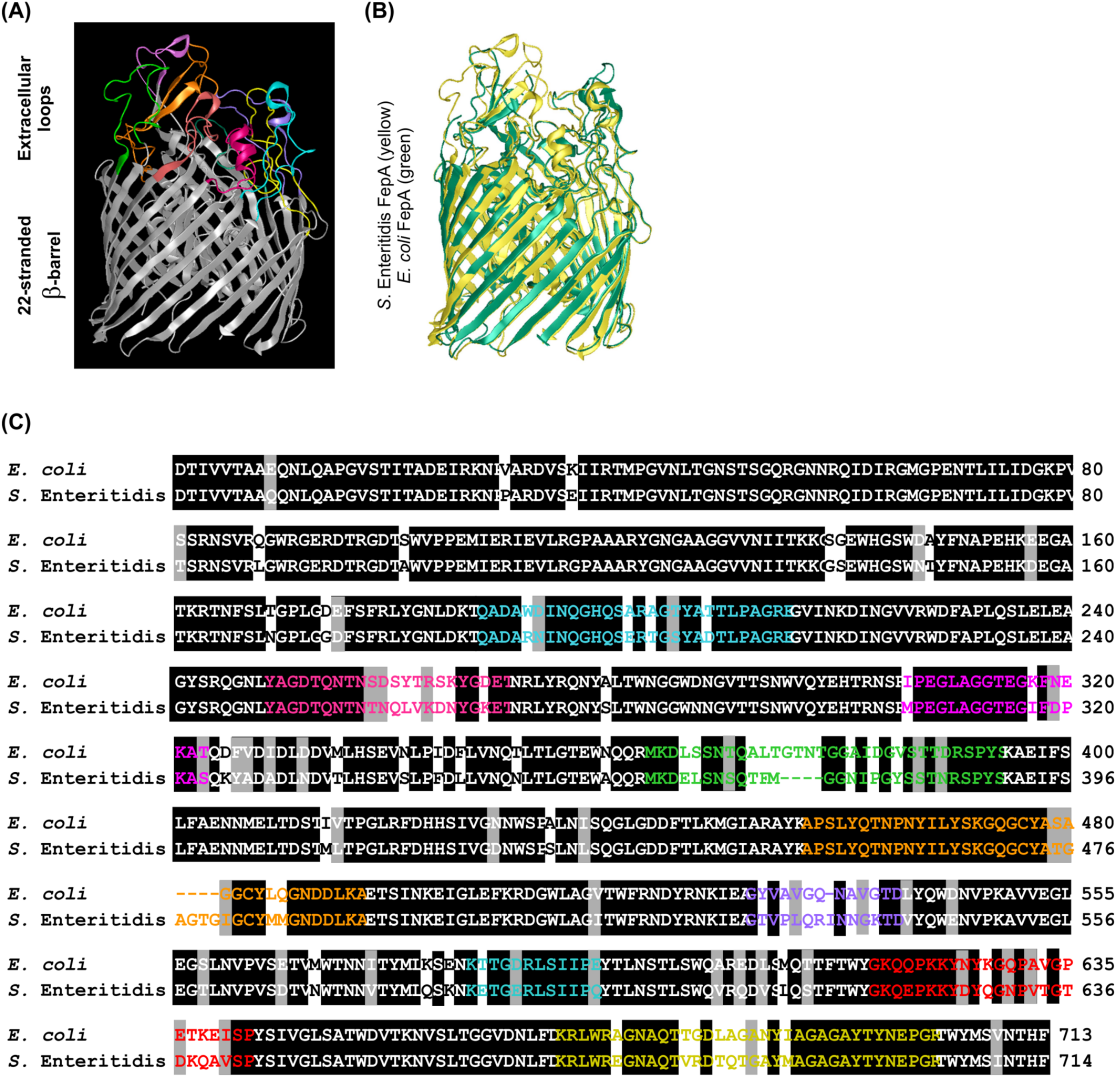
Prediction and comparison of *S.* Enteritidis FepA with *E. coli* FepA. **(A)** Predicted *S.* Enteritidis FepA homology model generated using the Phyre2 server. The figure depicts the extracellular space at the top and the periplasmic space at the bottom. The 22-stranded β-barrel, mainly embedded in the membrane bilayer, is represented in gray. The N-terminal domain is located within the β barrel channel. **(B)** Comparison of *S.* Enteritidis FepA (in yellow) with its homolog from *E. coli* (in green) through structural superimposition. **(C)** Sequence alignment of *S.* Enteritidis and *E. coli* FepA. Identical regions, mainly the 22-stranded β-barrel and the N-terminal domain within the channel, are shown in black boxes. Antigenic peptides, corresponding to extracellular loops, are shown in colored letters.

The nucleotide sequence of FepA was retrieved from *Salmonella enterica* subsp. enterica serovar Enteritidis str. P125109 complete genome (GenBank Accession Number AM933172.1). Homology models for this protein were predicted using the Phyre2 server. DNAStar software, Protean 3D package (DNASTAR, Inc., Madison, WI, USA), was used to predict *Salmonella* FepA antigenic peptides, employing criteria such as sequence length, hydrophilicity/hydrophobicity, surface orientation, and flexibility, along with B-cell epitope, T-cell epitope, and major histocompatibility class II (MHC II) epitope predictions. The predicted structures were overlaid on the solved structure of *Escherichia coli* FepA using Swiss PDB viewer, version 4.10 software (Guex and Peitsch, 1997) (**Figure 1****).** The amino acid sequence of FepA from *S.* Enteritidis and *E. coli* were aligned using BLASTp (https://blast.ncbi.nlm.nih.gov) to investigate whether the predicted antigenic peptides are present within the domain regions of other proteins and across different bacterial strains. This analysis aimed to identify conserved antigenic determinants that may be shared among various proteins and bacterial species, thereby providing insights into potential cross-reactivity. By comparing the peptide sequences against a comprehensive protein database, we sought to understand the distribution and conservation of these epitopes in a wider biological context.

**Figure 2:**
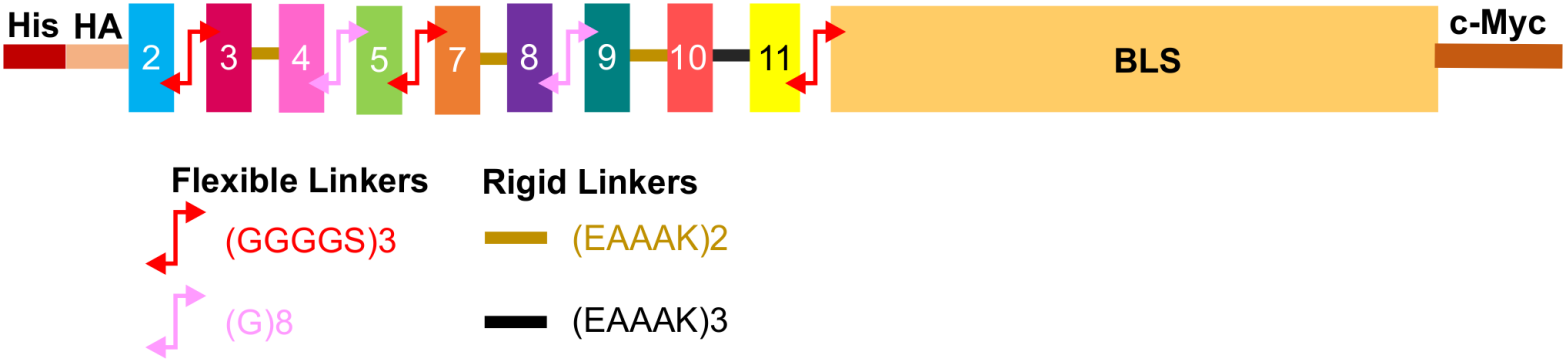
Vaccine candidate design. The *S.* Enteritidis FepA antigenic peptides, color-coded based on their respective loops, were connected to each other and to the N-terminus of *Brucella* lumazine synthase (BLS; PDB 1T13) using flexible and rigid linkers. BLS is depicted in light orange. A c-Myc tag is included at the C-terminus and an HA and His tag are included at the N-terminus for detection and purification.

The predicted surface-exposed loops of *S. Enteritidis* FepA were fused in tandem and to the N-terminus of BLS (PDB 1T13) using strategically chosen linkers to preserve epitope accessibility and structural independence. Flexible linkers such as (GGGGS)_2_ and (G)_8_ were used to minimize steric hindrance and allow conformational freedom, while rigid linkers like (EAAAK) and (EAAAK)_2_ provided spatial separation between adjacent peptides to reduce potential interference. This design ensured that the predicted extracellular loops were presented in a multivalent, immunologically relevant format. The linkers and their location in the construct are shown in **Figure 2**. The designed fusion construct was named FR-BLS.

### Cloning and transient expression in *N. benthamiana*

The designed construct was synthesized by Bio Basic Inc. (Mississauga, ON, Canada) with codon optimization for tobacco nuclear expression and incorporated flanking *Bsa*I recombination sites for subsequent Golden Gate cloning (Marillonnet and Werner, 2015) into plant expression vectors designed to target five distinct subcellular compartments. These vectors, derived from pCaMGate (Pereira et al., 2014) and referred to as pCLGGX (Vanderburgt et al., 2023), contain the double-enhanced constitutive Cauliflower Mosaic Virus 35S promoter and Nopaline synthase terminator for precise gene expression control. Expression vectors were transformed into *Agrobacterium tumefaciens* EHA105 via electroporation.

*N. benthamiana* served as the expression platform and were cultivated at a temperature of 22°C with a humidity level of 65%, under a 16:8 hour light-to-dark cycle within a walk-in growth chamber. The plants were grown in 4-inch pots filled with PRO-MIX BX soil and received appropriate watering supplemented with a 20:8:20 fertilizer (N:P:K) at a concentration of 2.5 g/L of water.

*A. tumefaciens* cultures carrying either the FR-BLS expression vector or a p19 expression vector, were cultured separately before being combined (each individual culture was diluted to a final OD_600_ = 0.5) and infiltrated into leaves of seven week old plants either by injection or vacuum, depending on the scale of production. p19 is a suppressor of post-transcriptional gene silencing from the cymbidium ringspot tombusvirus resulting in enhanced target protein expression (Silhavy et al., 2002). Sampling at 4, 6, and 8 days post-infiltration allowed for the validation of recombinant expression. Negative controls consisted of plants infiltrated solely with the p19 expression vector (OD_600_ = 0.5). Infiltration experiments were repeated at least three times.

### Protein extraction

Five plants were infiltrated and three leaf discs were collected from each plant, pooled, and analyzed. Pre-weighed leaf discs were flash frozen in liquid nitrogen and pulverized into a powder with silica beads (Bio Spec Products Inc., Bartlesville, OK, USA) using TissueLyser II (Qiagen, Venlo, Netherlands) for 2 minutes at 30 Hz. The resulting powder was then centrifuged at 20, 000 x *g* for 5 minutes and mixed well with protein extraction buffer (1x PBS pH 7.5, 0.1% Tween-20 (v:v), 2% polyvinylpolypyrrolidone (PVPP) (v:v), 1 mM EDTA pH 8.0, 1 mM phenylmethylsulfonyl fluoride (PMSF), 1 µg/ml Leupeptin, and 100 mM Sodium L-ascorbate) in a 1:3 weight/volume ratio. All samples were vortexed at high speed for 30 s and sonicated with a 30-s burst at 30% amplitude (Fisherbrand™ Model 120 Sonic Dismembrator, Fisher Scientific, Schwerte, Germany). The extracts were centrifuged at 20, 000 x *g* for 5 min at 4°C, and the supernatants containing total soluble proteins (TSP) were recovered.

### Western blot analysis

The extracted proteins underwent denaturation by mixing with reducing loading buffer (0.3 M Tris-HCl pH 8.0, 5% SDS (w:v), 10% glycerol (w:v), 100 mM DTT, 0.05% Phenol Red) and were heated at 100°C for 10 minutes. Subsequently, 20 µl of each protein sample were loaded onto Express Plus 4-20% gradient polyacrylamide gels (Genscript Inc., Piscataway, NJ, USA) and subjected to electrophoresis at 100 V for 100 minutes. Following electrophoresis, the proteins were transferred onto polyvinylidene difluoride (PVDF) membranes using the Trans-Blot Turbo transfer system (Bio-Rad Laboratories Inc., Hercules, CA, USA). These membranes were then blocked overnight at 4 °C in a 5% blocking solution (5% skimmed milk in Tris-buffered saline (TBS; 20 mM Tris, 150 mM NaCl, pH 7.5)). Immunodetection involved probing the proteins with one of the following primary antibodies, mouse anti-hemagglutinin (HA) (Sigma-Aldrich Cat. No. H3663), mouse anti-c-Myc (GenScript Cat. No. A00864), or mouse anti-His (Takara Bio USA Inc. Cat. No. 631212), each used individually at a 1:5, 000 dilution in 0.5% blocking solution for 1 hour at room temperature. After three washes in TBS pH 7.5, the membranes were incubated with goat anti-mouse horseradish peroxidase (HRP)-conjugated antibodies (Bio-Rad <ns/>1706516), diluted 1:5, 000 in 0.5% blocking solution. Chemiluminescent signals were detected using Enhanced Chemiluminescent detection solution (Bio-Rad Laboratories Inc., Hercules, CA, USA) and imaged with a MicroChemi imaging system using GelCapture acquisition software (DNR Bio-Imaging Systems Ltd., Jerusalem, Israel). Additionally, proteins separated by SDS-PAGE were stained using GelCode^®^ Blue Stain Reagent solution (Thermo Fisher Scientific Inc., Waltham, MA, USA), followed by destaining with ultrapure water.

### Enzyme-linked immunosorbent assay (ELISA)

ELISA MaxiSorp 96-well plates (Thermo Fisher Scientific Inc., Waltham, MA, USA) were coated with in-house rabbit anti-BLS antibody (1:1, 000 dilution in 0.1 M NaCO_3_/NaHCO_3_, pH 9.4) at 4 °C overnight, followed by blocking with 2% Bovine Serum Albumin in Phosphate-Buffered Saline (BSA-PBS) at room temperature. After washing, plates were incubated with serially diluted protein extracts in 1% BSA-PBS overnight at 4 °C and subsequently probed with mouse anti-HA primary antibody (1:5, 000) for 1 hour at room temperature. After washing, goat anti-mouse Horseradish Peroxidase (HRP)-conjugated secondary antibody (1:5, 000) was applied for 1 hour at room temperature. Following additional washing steps, antigen-bound antibodies were detected using 2, 2’-azino-bis (3-ethylbenzothiazoline-6-sulfonic acid (ABTS, Sigma Cat. No. A1888), and the colorimetric reaction was measured at 415 nm using a plate reader (iMark™ Microplate Absorbance Reader, Bio-Rad, Feldkirchen, Germany), with negative control samples consisting of protein extracts from plants agroinfiltrated with only p19, as well as blank wells containing coating buffer and blocking solution only, and no primary antibody controls, in which samples were added but the primary antibody step was omitted. Coating ELISA plates with BLS antibodies allowed us to capture the FR-BLS construct specifically, enhancing the accuracy of the quantification process.

### Recombinant protein purification

Recombinant proteins were purified using immobilized metal affinity chromatography (IMAC). Leaves were ground in liquid nitrogen in the presence of protein extraction buffer containing 20 mM imidazole (1:3 weight/volume ratio) and sonicated with a 30-second burst at 40% amplitude for 3 minutes (Fisherbrand™ Model 120 Sonic Dismembrator, Fisher Scientific, Schwerte, Germany). After filtration through miracloth and centrifugation at 20, 000 x *g* for 20 minutes, the supernatant was mixed with Ni Sepharose 6 Fast Flow histidine-tagged protein purification resin (Cytiva, Cat. No. 17531801) and incubated on a shaker for 1 h at 4°C. This step was followed by centrifugation at 1, 000 × *g* for 2 min at 4 °C. The flow-thru supernatant was discarded, and the remaining beads were washed with five-resin volumes of wash buffer (PBS containing 20 mM imidazole) 3 times (1,000 × *g* for 2 min at 4°C). The Ni^2+^ resin was then packed in a column (the resin volume after packing was about 500 µl) and the recombinant protein was eluted with five-resin column volumes of elution buffer (PBS containing 500 mM imidazole). Five fractions, each approximately 500 µl, were collected. IMAC-purified proteins were further purified by size-exclusion chromatography (SEC) on a Superose 6 Increase 10/300 GL column (Bio-Rad Laboratories Inc., Hercules, U.S.A.) equilibrated in PBS buffer.

### Chickens, housing and study design

An immunogenicity study of the designed FR-BLS was conducted at the Arkell Poultry Research Station and approved by the University of Guelph Animal Ethics Committee (Animal User Protocol No.3521), according to the Canadian Council of Animal Care guidelines (CCAC 2009). A total of 60 male Ross 708 day-old broiler chicks were weighed individually and distributed between 10 identical cages (6 birds/cage). Heat, water, and feed were provided according to Ross 708 broiler performance objectives (Aviagen, 2022). Different diets were administered based on age (starter, grower, finisher), tailored to meet the broilers’ nutritional requirements (**Table 1**). The study included five cages per group (30 birds/treatment), with the FR-BLS group receiving intramuscular vaccinations (50 µg/bird) of FR-BLS with Incomplete Freund’s adjuvant on day 1 and a booster dose on day 21, while the control group received 1X PBS (Kaneshige et al., 2009; Kithama et al., 2022; Fruci et al., 2023).

**Table 1:** The composition of the starter (days 0 - 14), grower (days 15 -28), and finisher (days 29 - 35) diets used in this study.

Ingredient, %	Starter	Grower	Finisher
Corn	42.1	44.8	49.2
Wheat	10.0	10.0	10.0
Soybean meal	38.5	35.1	30.1
L-Lysine HCL	0.28	0.22	0.21
DL-Methionine	0.35	0.30	0.28
L-Threonine	0.15	0.10	0.09
Limestone Fine	0.97	0.86	0.77
Monocalcium phosphate	2.11	1.89	1.69
Salt	0.23	0.25	0.25
Sodium bicarbonate	0.21	0.18	0.18
Vitamins and trace minerals premix^1^	0.50	0.50	0.50
Soy oil	4.43	5.64	6.56
Monteban (Narasin Type A)	0.07	0.07	0.07
Bacitracin Methylene Disalicylate (BMD)	0.05	0.05	0.05
Ingredient Total:	100	100	100

^1^Provided per kilogram of diet: vitamin A, 8800.0 IU; vitamin D3, 3300.0 IU; vitamin E, 40.0 IU; vitamin B12, 12.0 mg; vitamin K3, 3.3 mg; niacin, 50.0 mg; choline, 1200.0 mg; folic acid, 1.0 mg; biotin, 0.22 mg; pyridoxine, 3.3 mg; thiamine, 4.0 mg; calcium pantothenic acid, 15.0 mg; riboflavin, 8.0 mg; manganese, 70.0 mg; zinc, 70.0 mg; iron, 60.0 mg; iodine, 1.0 mg; copper, 10 mg; and selenium, 0.3 mg.

### Data collection

Data were collected as previously described (Das et al., 2020; Kithama et al., 2022; Fruci et al., 2023). Chicks were weighed at the start of the trial (day 0) and every week thereafter. Performance parameters including bodyweight (BW), feed intake (FI) and feed conversion ratio (FCR) were assessed on days 14 (starter phase), 28 (grower phase), and 35 (finisher phase) for each cage. On days 21, 28, and 35, two birds per cage (10 birds per treatment) were euthanized by cervical dislocation (Das et al., 2020; Fruci et al., 2023) to collect blood for antibody titer (ELISA) determination, and immune organs (spleen, liver, and bursa) were weighed and preserved for further analysis. Immune−organ weights were normalized to body weight to ensure valid comparisons.

### Serum antibody titers

Serum samples collected on days 21, 28, and 35 were analyzed to assess general IgA, IgY, and IgM antibody levels and FepA-specific IgA, IgY, and IgM antibody titers (Ster et al., 2010). General IgA, IgY, and IgM antibody levels were quantified using ELISA kits (Bethyl Laboratories) as per the provided protocol. To quantify FepA-specific IgA, IgY, and IgM antibody levels, Nunc MaxiSorp 96-well plates (Thermo Fisher Scientific Inc., Rochester, NY, USA) were coated with FepA predicted antigenic peptides (L2, L3, L4, L8, and L10) fused to encapsulin, another protein nanoparticle, at 5 µg/ml in 0.05 M carbonate/bicarbonate buffer, incubated overnight at 37°C, and then blocked with a PBS-based solution containing 3% skim milk powder for 1 hour at 37°C. Sera, diluted at 1:175, were added to the plates (100 µl) and incubated for 1 hour at 37°C. Following three washes with PBS containing 0.05% Tween 20, 100 µl of HRP-conjugated secondary antibody (goat anti-chicken IgG, IgA, and IgM HRP) diluted at 1/10, 000 in blocking solution (Abcam, Cambridge, United Kingdom) was added to each well. The plates underwent incubation with TMB substrate (Bethyl Laboratories) in the dark at room temperature for 30 minutes, followed by termination of the reaction using ELISA stop solution (Bethyl Laboratories). ELISA plate readings at 450 nm were conducted using the BioTek PowerWave XS2 plate reader.

### RNA isolation from bursa

To evaluate the immunomodulatory effect of the vaccine on immune-gene expression in broilers, the collected bursal tissue samples harvested from FR-BLS-vaccinated (n=10) and control (n=10) birds at 35 days of age were investigated. Using sterile forceps and scissors, the entire immune organs were removed from the carcass, stabilized immediately in RNA stabilization solution (AM7021, ThermoFisher Scientific) for gene expression profiling (1 mg tissue sample to 1 mL stabilization solution ratio). The submerged tissue samples were snap-frozen and stored at −80°C until further analysis. Samples of RNA were extracted following the procedures previously described (Das et al., 2020).

### Gene expression analysis by quantitative PCR

cDNA synthesis and quantitative PCR were performed as per Qiagen RT2 Profiler PCR Array Handbook 11/2018 (Das et al., 2020). In brief, 2 mg of total RNA was subjected to cDNA synthesis using the RT2 First Strand Kit (Qiagen, Valencia, CA). For each sample, cDNA was mixed with molecular-grade water and RT2 SYBR Green ROX quantitative Polymerase Chain Reaction Master Mix (Qiagen, Valencia, CA) and added to each well of a 96-well plate from the Chicken Innate & Adaptive Immune Response PCR Array (PAGG-052ZA, Qiagen). These plates were used to profile the expression of 84 genes functionally grouped into innate, adaptive, humoral immunity, inflammatory response, and defense response against bacteria and viruses as described by Das et al. (2020). Five genes (*ACTB*, *H6PD*, *HMBS*, *RPL4*, and *UBC*) were provided as housekeeping genes. In addition, the last seven genes were used as internal controls (genomic DNA contamination, reverse transcription control, and general PCR performance). Real-time PCR was performed using an Applied Biosystems 7500 Real-time PCR System with 7500 Software v 2.3. To ensure accurate comparisons between curves, the same threshold was applied for all genes and samples during analysis. Gene expression was normalized with five housekeeping genes *IFNGR1*, *TLR7*, *CASP8*, *STAT6* and *TLR2B*, automatically selected by RT2 Profiler PCR Arrays & Assays Data Analysis (Qiagen). Fold changes in gene expression between control and the vaccinated group were calculated using the 2^−ΔΔCt^ method.

### Statistical analyses

Statistical analyses on growth performances and serum Ig levels were conducted according to a randomized complete block design using the General Linear Mixed Model (GLMM) procedure of the Statistical Analysis System, version 9.4 (SAS Institute Inc., Cary NC). Vaccination and age (day of sampling) were used as sources of variation and the individual cage as experimental units (five cages/vaccination group). Least significance difference was used to separate means whenever the F-value was significant. The difference in fold changes (FCs) of gene expression between control and vaccinated groups was estimated using the 2^−ΔΔCt^ method and the Student’s t-test. ǀFCǀ ≥ 2 were considered as significantly influenced by feed supplementation. The *P*-value of 0.05 was used to determine statistical significance.

## Results

### Vaccine candidate design

There is no available crystal structure of FepA from *S.* Enteritidis. To identify possible antigenic peptides, a 3-dimensional (3D) homology model for *S.* Enteritidis FepA, was predicted using Phyre2 (Kelley et al., 2015) (**Figure 1**). Phyre2 showed 100% confidence in the overall architecture of the model. The 22-stranded β-barrel structure extends across the outer membrane, with most of these strands protruding into the extracellular space. These strands are intricately linked through loops on the extracellular side and brief turns on the periplasmic side (**Figure 1**).

The superimposition (overlay) of the predicted structure of *S.* Enteritidis FepA on the related structure from *E. coli* (Buchanan et al., 1999), coupled with the amino acid sequence alignment of FepA from *S.* Enteritidis and *E. coli* (**Figures 1B**, C), highlighted resemblance in the 22-stranded β-barrel structure across the outer membrane, similar to other TonB-dependent transporters. The disparity between *S.* Enteritidis FepA and *E. coli* FepA primarily resides in the extracellular loops (**Figures 1B**, C). This distinction holds critical importance as it suggests that a vaccine candidate engineered to target these extracellular loops could specifically impact *S.* Enteritidis, the intended microbial target, without affecting its counterpart in *E. coli*.

The antigenic peptides of *S.* Enteritidis FepA, predicted using the DNAStar software, Protean 3D package, typically fell within the 10–20 residue range, balancing immunogenicity and specificity (Singh and Mishra, 2016; Bojarska and Wolf, 2024). Antigens that were shorter than 10 residues (L1 and L6) were ignored. However, those longer than 20 residues were included in the design of the candidate vaccine construct. Identified within the protein’s hydrophilic, flexible, and surface-oriented extracellular loops, these antigenic peptides conformed to epitope characteristics associated with surface mobility and accessibility and also exhibited the distinct features of B-cell epitopes, T-cell epitopes, and MHC II epitopes. The anticipated antigenic peptides were labeled with “L” representing ‘Loop’, followed by a numerical identifier corresponding to their specific order within the loops.

To verify if the sequences of the identified antigenic peptides are found in other Salmonella serovars, we searched the protein database with BLASTp. The antigenic peptide sequences were found with 100% identity in FepA from all *Salmonella* serovars. This finding indicates that a vaccine candidate containing these antigens may be effective against most *Salmonella* serovars.

To simultaneously present all predicted antigenic peptides to the immune system and enhance their immunogenicity, these peptides were genetically fused in tandem to the N-terminus of BLS using both flexible and rigid linkers to produce the chimeric protein called FR-BLS (**Figure 2**). Different constructs using various linkers were designed, and their structures were predicted *in silico*. Three constructs that showed the peptides as more accessible were selected and ordered for gene synthesis. Based on the accumulation results of these constructs in *N. benthamiana*, FR-BLS was chosen for further studies.

### Vaccine candidate expression, purification, and assembly analysis

**Figure 3:**
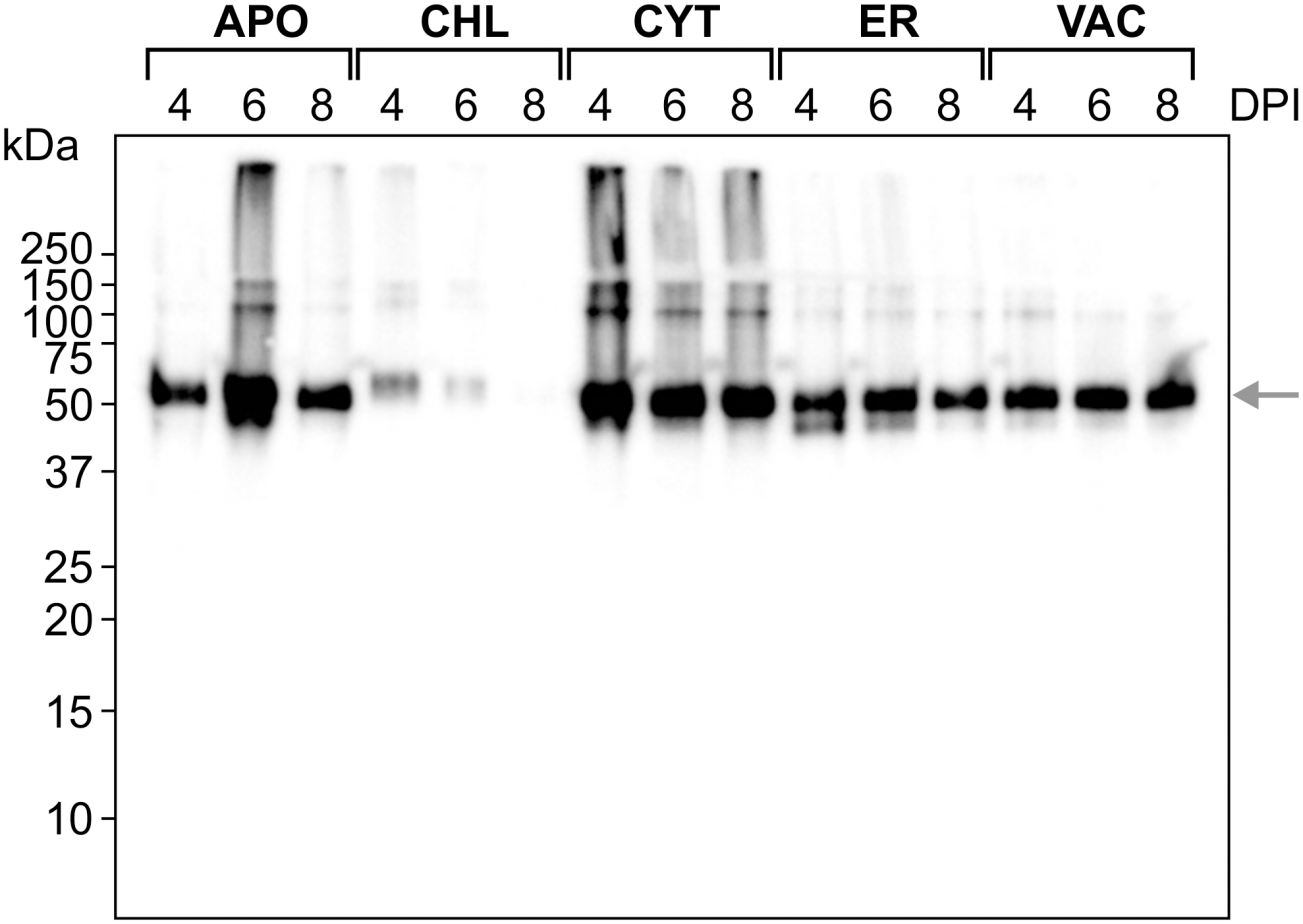
Expression of FR-BLS in *N. benthamiana.* FR-BLS was expressed in five distinct subcellular compartments of *N. benthamiana*. Tissues were collected at 4-, 6-, and 8-days post-infiltration (DPI). Twenty µl of protein sample was loaded in each well. An anti c-Myc antibody (1:5,000) was used to detect the protein. The target protein, FR-BLS, approximately 52 kDa, is indicated by the green arrow. APO, Apoplast; CHL, chloroplast; CYT, cytosol; ER, endoplasmic reticulum; VAC, vacuole. DPI, days post infiltration.

FR-BLS was targeted to various subcellular compartments within *N. benthamiana* and immunoblot analysis of whole cell lysates revealed that protein accumulation was highest in the cytosol (**Figure 3**). As both *Salmonella* and *Brucella* are prokaryotes, their proteins are not glycosylated, and the cytosol is a desirable compartment for accumulating FR-BLS. Cytosol-targeted FR-BLS accumulated to approximately 0.22 mg/g of fresh plant weight eight days post-infiltration. In the industrial manufacturing of pharmaceutical proteins derived from plants for human use, the assessment of economic viability relies on an informal benchmark of 1% of the total soluble protein, or roughly 0.1 mg/g of recombinant protein per fresh plant weight (Rybicki, 2009).

**Figure 4:**
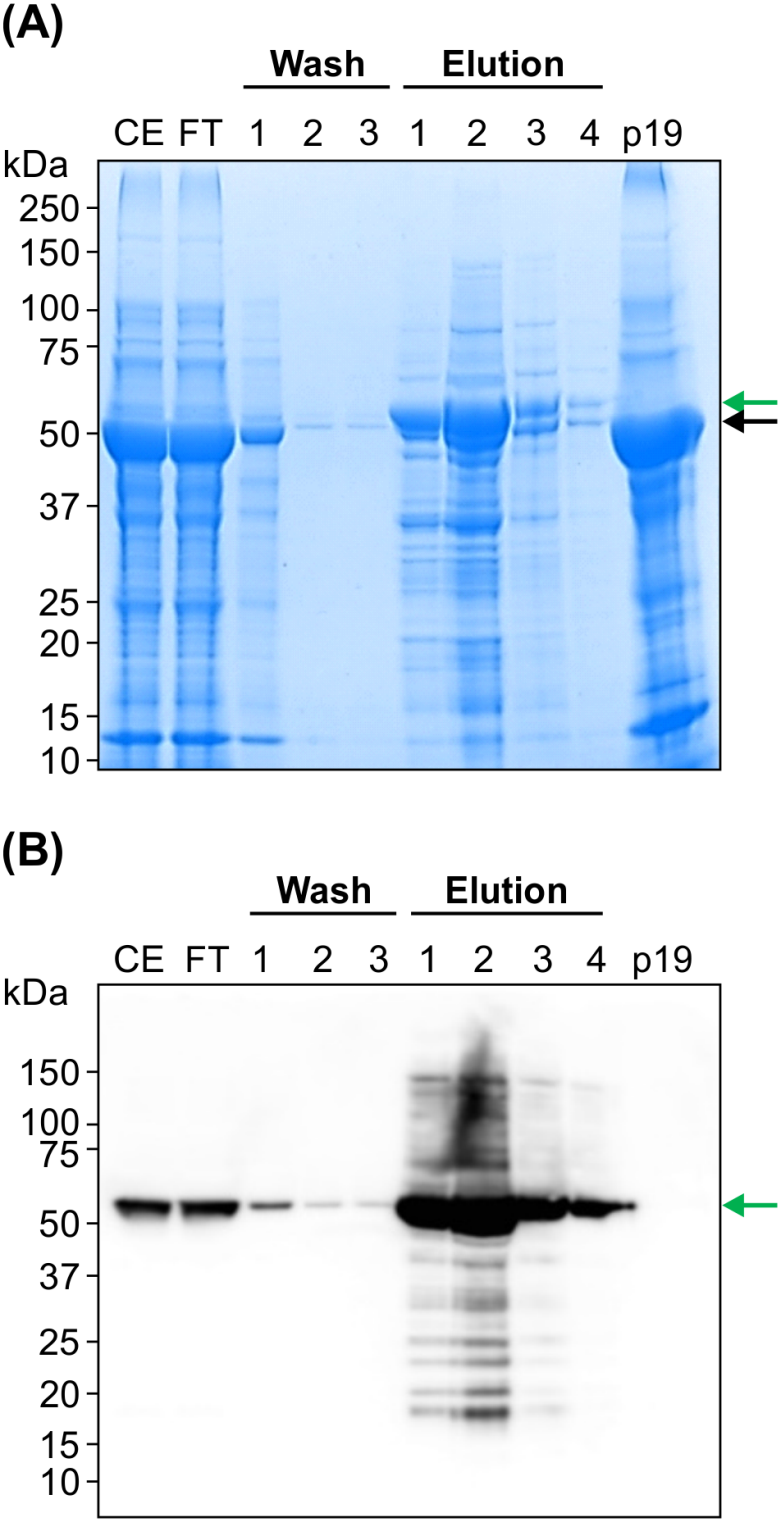
FR-BLS is concentrated and partially purified by IMAC. **(A)** IMAC-purified FR-BLS was analyzed by SDS-PAGE. The size of FR-BLS (green arrow) is slightly bigger than the large subunit of RUBisCO (RBC-L; black arrow). **(B)** Specific detection of FR-BLS with an anti-His-tag antibody (1:5,000) and western blotting. 5 µl of sample was loaded in each well. CE: crude extract; FT, Flow through; p19, negative control.

FR-BLS was then enriched using immobilized metal affinity chromatography (IMAC). The size of denatured monomeric FR-BLS (52 kDa) as visualized via SDS-PAGE is very close to that of the large subunit of ribulose bisphosphate carboxylase (RBC-L), an abundantly produced enzyme in *N. benthamiana*. FR-BLS migrated slightly higher than RBC-L following IMAC enrichment as observed via SDS-PAGE and immunoblot analysis (**Figure 4**).

**Figure 5:**
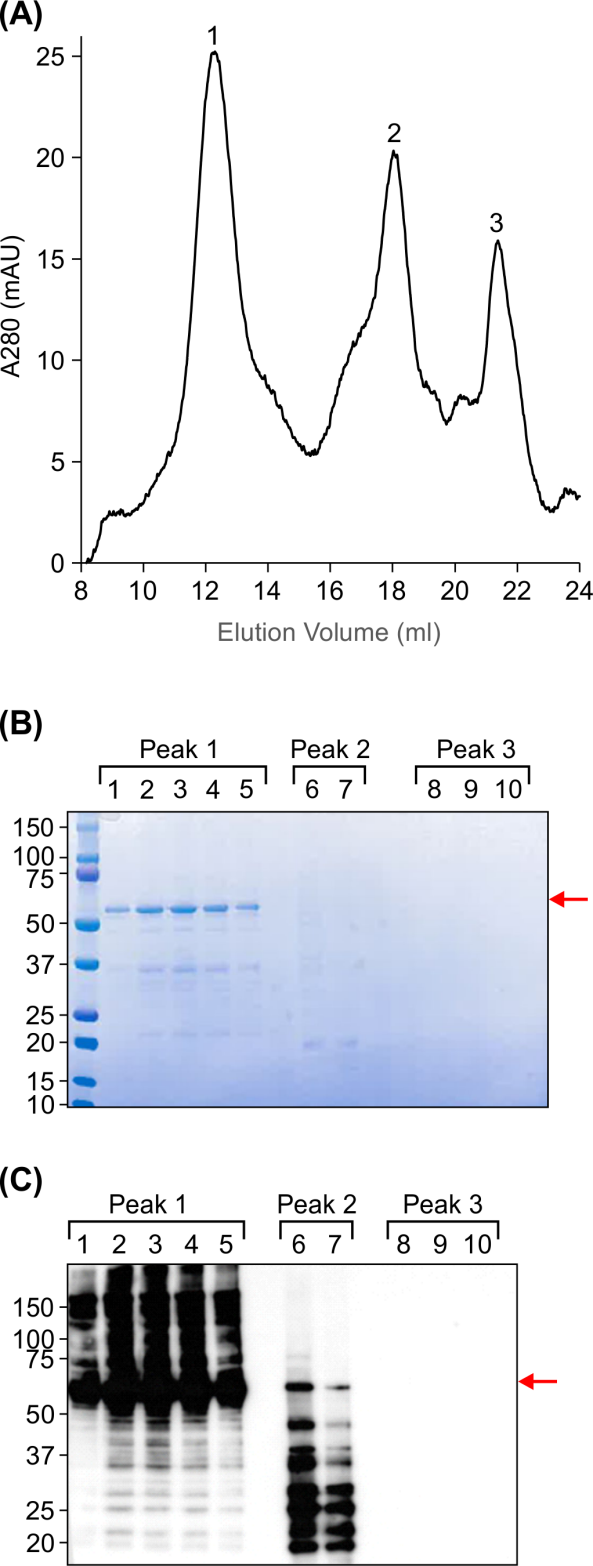
FR-BLS assembles into nanoparticles. **(A)** Size exclusion chromatogram showing three distinct peaks, labelled 1, 2 and 3. **(B)** SDS-PAGE analysis of fractions collected under the three peaks showed a prominent 52 kDa band corresponding to FR-BLS in peak 1, which eluted at 12.5 ml, indicative of nanoparticle assembly. **(C)** Immunoblotting reveals the presence of 52 kDa FR-BLS (black arrow) in peaks 1 and 2. Bands with higher and smaller molecular weights detected on the immunoblot may stem from incomplete denaturation of assembled particles and degradation of the FR-BLS protein, respectively. Peak 3 showed no FR-BLS signal. Red arrow points to the FR-BLS band.

Following the initial IMAC enrichment, calibrated size exclusion chromatography (SEC) revealed the formation of FR-BLS decameric structures. Three distinct peaks were observed on the chromatogram, suggesting a heterogeneous population of particles, with the highest peak eluting at 12.5 ml from a Superose 6 Increase 10/300 GL SEC column (**Figure 5A**). According to the SEC column user manual, another protein nanoparticle, Ferritin (440 kDa) elutes at 15 ml. The molecular weight of an assembled decameric FR-BLS nanoparticle is about 520 kDa, and is expected to elute earlier than 15 ml. Therefore, the major peak appears to represent an assembled nanoparticle. The second peak eluted at about 18 ml, in the same range as Ovalbumin (44 kDa), which would represent a monomeric FR-BLS protein. The third peak at 22 ml might contain small molecules unrelated to FR-BLS. To examine the identity of the proteins eluting at these peaks, eluted fractions that fell under the peaks were analyzed by SDS-PAGE (**Figure 5B**) and Western blotting (**Figure 5C**). Most FR-BLS protein was found in fractions eluted from the first peak at 12.5 mL, with multiple bands of higher molecular weight observed on the immunoblot. These bands may be due to incomplete denaturation of assembled decameric particles. A band the size of full-length FR-BLS was observed in fractions corresponding to the peak at 18 mL, indicating the presence of monomeric BLS. However, in those fractions, no larger bands were observed, and several smaller bands were apparent which may indicate degradation of FR-BLS. Fractions collected from the peak at 22 mL did not contain any FR-BLS.

### Safety of the FR-BLS vaccine candidate

An immunization study was conducted to evaluate the immunogenicity of FR-BLS in broiler chickens. Day old chicks were immunized with IMAC-purified FR-BLS by intramuscular injection on day 1 and day 21. To evaluate the safety of the vaccine candidate, growth performance metrics such as bodyweight, feed intake, and feed conversion ratio were evaluated (**Table 2**). No statistically significant differences (*P* > 0.05) were observed between the FR-BLS and control groups (**Table 2**). The assessment of immune organ weight is also a critical indicator of immune system development (Choi et al., 2021; Song et al., 2021). At days 21, 28 and 35 of age, two birds/cage (10 birds/treatment) were sacrificed to collect blood for antibody titers (ELISA) and immune organs (spleen, liver, and bursa). No significant treatment effects were noted in the weights of immune organs between the FR-BLS and control groups (**Table 3**).

**Table 2:** Effects of FR-BLS on growth performances in broilers. *P*-value was obtained by ANOVA.

Parameters	Treatments		
BW (Kg/bird)	Control	FR-BLS	*P*-value
d 0	0.04	0.04	0.65
d 14	0.32	0.33	0.81
d 28	1.33	1.31	0.52
d 35	2.11	2.00	0.31
FI (Kg/bird)			
d 0-14	0.55	0.55	0.68
d 15-28	1.48	1.47	0.82
d 29-35	1.24	1.16	0.17
d 0-35	3.26	3.18	0.34
FCR			
d 0-14	1.95	1.93	0.81
d 15-28	1.94	1.82	0.29
d 29-35	1.51	1.54	0.63
d 0-35	1.65	1.60	0.90

BW, body weight; FI, feed intake; FCR, feed conversion ratio.

**Table 3:** Effect of FR-BLS on immune organs (liver, spleen, bursa) indices in broilers.

Day	Index	Treatments		*P*-value
	(Organ/BW) (g/kg)	FR-BLS	Control	
21	Liver	30.4	30.1	0.78
	Spleen	0.86	0.85	0.94
	Bursa	2.30	1.94	0.35
28	Liver	27.5	27.4	0.93
	Spleen	0.94	0.92	0.78
	Bursa	2.06	2.04	0.91
35	Liver	24.1	23.5	0.62
	Spleen	0.99	1.12	0.37
	Bursa	1.67	1.56	0.66

*P*-value was obtained by ANOVA.

### General immunoglobulins

**Figure 6:**
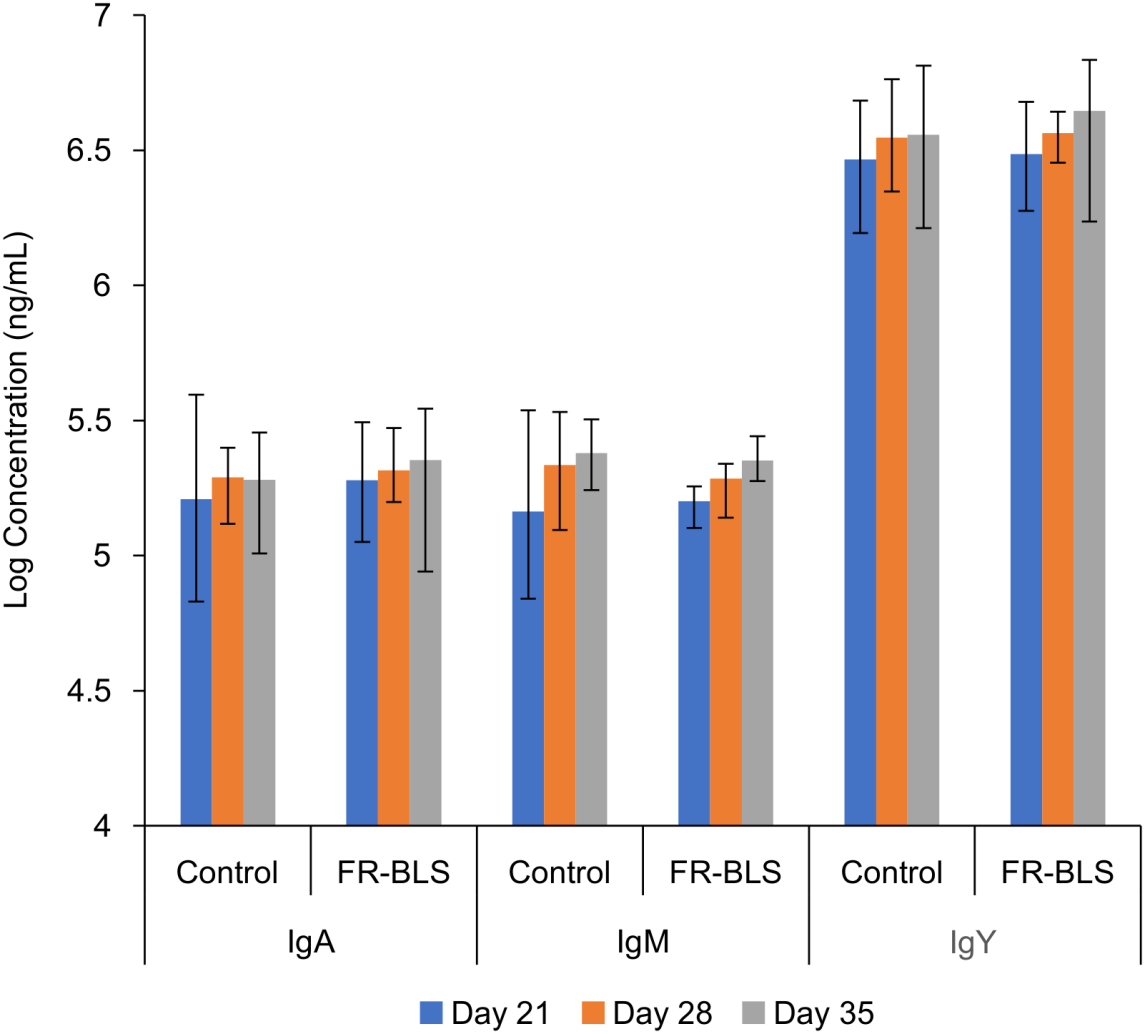
Overall levels of general immunoglobulins including IgA, IgM, and IgY antibodies were similar between immunized and control chickens. The experiment was comprised of five cages per group, each containing six birds (n = 30 per group). The control group received 1X PBS, and immunized chickens received 50 μg FR-BLS. Statistical analysis was performed using the general linear model (GLM) procedure of SAS (version 9.4, SAS Institute Inc, Cary, NC). The results are the means of the biological replicates. Error bars represent standard deviation of the mean. No significant differences in total general IgY, IgA and IgM were observed between the FR-BLS and control groups at all three days (*P* > 0.05).

Serum samples collected on days 21, 28, and 35 were analyzed to assess the overall levels of general immunoglobulins including IgA, IgY, and IgM. No significant differences (*P* > 0.05) in the titers of total general IgY, IgA and IgM were observed between the FR-BLS and control groups on all three days (**Figure 6**). This observation suggests that the birds’ immune system remained unaffected by the vaccination with FR-BLS.

### Specific IgY immune response to the vaccine candidate

**Figure 7:**
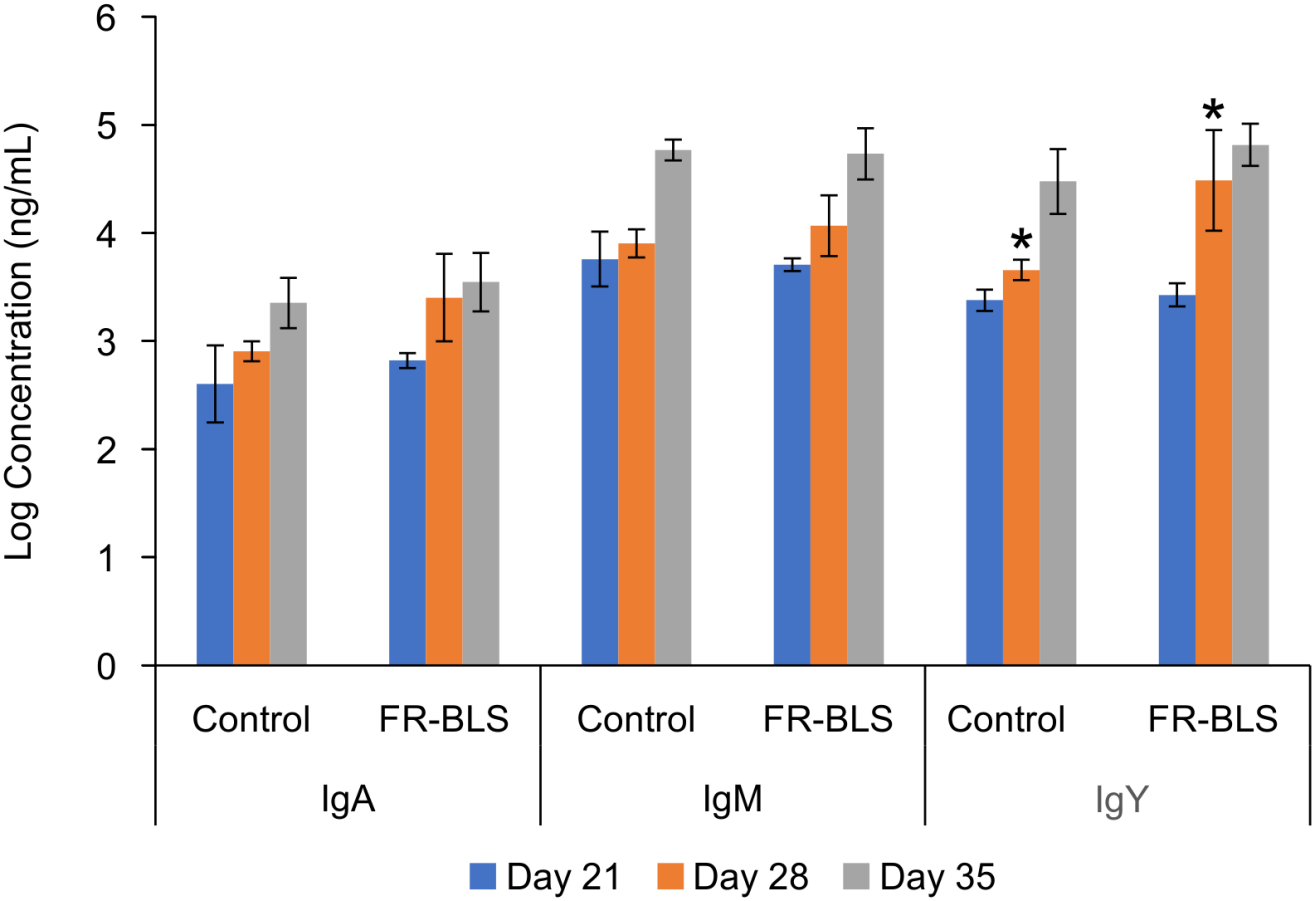
IgY antibodies specific to FepA antigenic peptides were detected at day 28 post-immunization. The presence of specific IgA, IgM, and IgY antibodies targeting FepA antigenic peptides following immunization with FR-BLS was examined and compared to a control group on days 21, 28, and 35. Each group consisted of five cages housing six birds each, with the control group receiving 1X PBS, and immunized chickens receiving 50 μg FR-BLS. ELISA plates were coated with FepA antigenic peptides (L2, L3, L4, L8, and L10) fused to encapsulin, another protein nanoparticle. The results are the means of the biological replicates Statistical analysis employed the general linear model (GLM) in SAS (version 9.4, SAS Institute Inc, Cary, NC), and error bars represent standard deviation of the mean. Asterisks indicate a significant difference in IgY levels on day 28 between the FR-BLS treatment and the control group (*P* < 0.05).

To ascertain that the immune response was specific to the *S.* Enteritidis FepA loops and not to BLS, serum samples collected on days 21, 28, and 35 were analyzed to assess FepA-specific IgA, IgY, and IgM antibody titers. Each antibody type plays a distinct role in immunity, with IgA safeguarding mucosal surfaces, IgY providing systemic protection, and IgM representing the initial response to antigens (Casali and Schettino, 1996; Daëron, 2024). All ELISAs were conducted with FepA antigenic peptides (L2, L3, L4, L8, and L10) displayed on encapsulin, a different protein nanoparticle. On day 28, the birds injected with FR-BLS exhibited significantly elevated levels of FepA-specific IgY antibodies compared to the control treatments, as indicated by asterisks (P < 0.05) (**Figure 7**). By day 35, the level of IgY antibodies in the controls increased and masked the effect of the immune response to FR-BLS. It is not clear why IgY antibodies recognizing *S.* Enteritidis FepA developed in the control groups on day 35, it may be that the birds encountered *Salmonella* during the experiment. On the other hand, no significant differences were found in IgA or IgM levels between the FR-BLS treatment and the control group (**Figure 7**), which is expected as the birds were immunized intramuscularly which would not trigger a strong mucosal immune response (Nochi et al., 2018; Thwaites et al., 2023). Despite the induction of antigen specific IgY responses, no changes in immune organ weights were detected, indicating that the vaccine elicited a focused humoral response without triggering broad lymphoid activation or systemic inflammation. This pattern is consistent with the presentation of defined linear epitopes on the BLS scaffold, which is expected to stimulate antigen specific antibody production without causing generalized immune organ enlargement.

### Expression of innate and adaptive immune genes by quantitative PCR array

**Figure 8:**
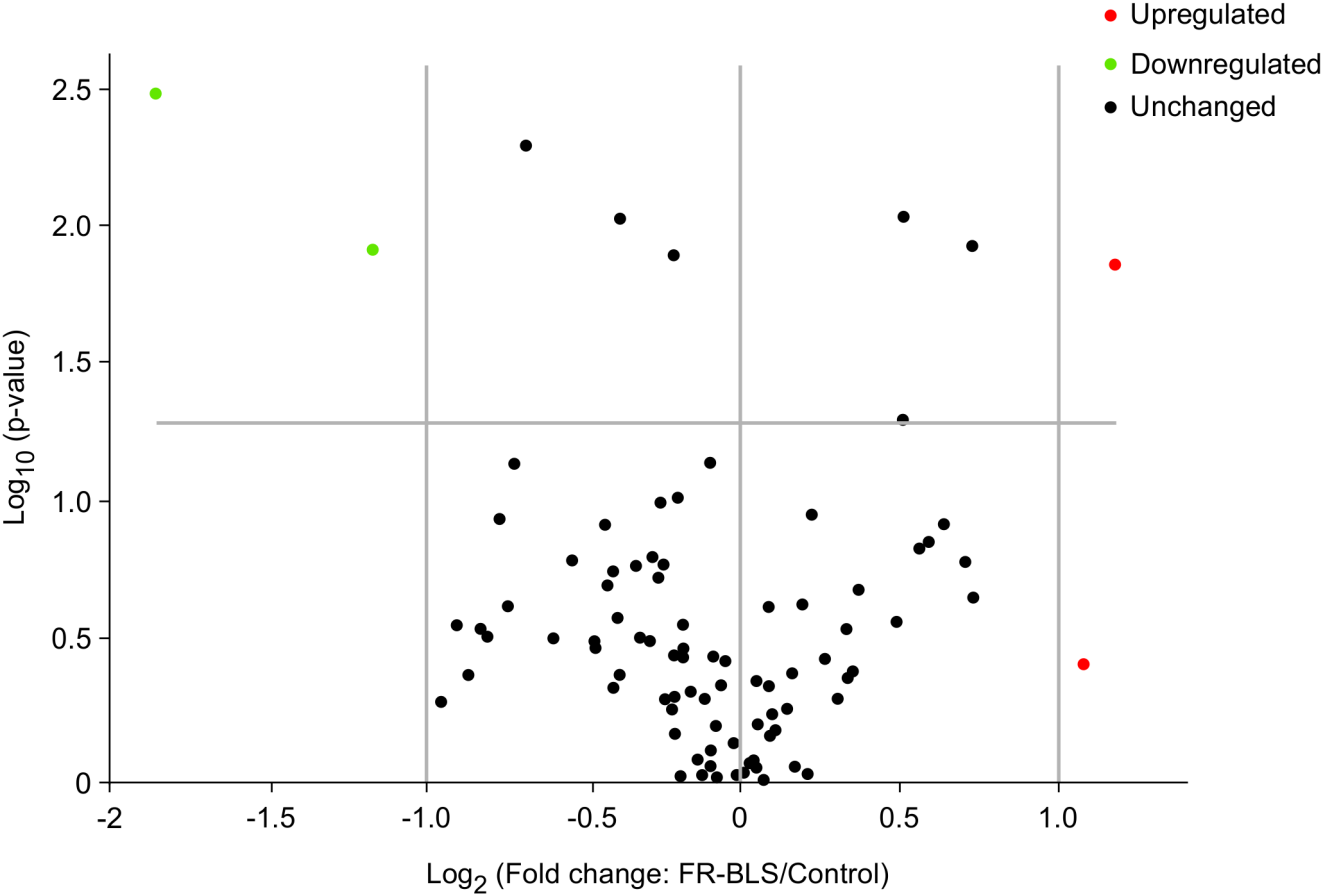
The expression of three genes in the chicken bursa was significantly altered in response to immunization with FR-BLS. Volcano Plot of the expression of innate and adaptive immune genes by quantitative PCR array. The center vertical line indicates unchanged gene expression, while the two outer vertical lines indicate the selected fold regulation threshold. The horizontal line indicates the selected *P*-value threshold. Genes with data points in the far upper left (down-regulated) and far upper right (up-regulated) sections meet the selected fold regulation and *P*-value thresholds.

To investigate the effects of FR-BLS vaccine on broiler immune gene expression, a qPCR array covering 84 immune-associated genes was performed. **Table 4** and **Figure 8** summarize the differentially expressed genes in the bursa of Fabricius of birds receiving the FR-BLS vaccine compared to the unvaccinated control birds. Overall, nine genes were differentially expressed in the bursa, with only three genes being significantly (*P* < 0.05) modulated above ǀFCǀ ≥ 2. The solute carrier family 11 (proton-coupled divalent metal ion transporters) member 1 (SLC11A1) gene was found to be upregulated (fold change = 2.26) in FR-BLS-vaccinated birds when compared to control. Significant downregulation in the expression of the chemokine (C-C motif) receptor 5 (CCR5, fold change = -2.24) and the actin, beta (ACTB, fold change = -3.61) genes were observed in FR-BLS-vaccinated birds (*P* < 0.05) compared to control.

**Table 4:** Genes that were significantly regulated in chicken bursa in response to by FR-BLS-vaccination compared to control non-vaccinated birds.

Gene	Description	Fold change	P-value
*SLC11A1*	Proton-coupled divalent metal ion transporters	2.26	0.013
*CCR4*	Chemokine (C-C motif) receptor 4	1.42	0.0089
*HMBS*	Hydroxymethylbilane synthase	1.65	0.0114
*IL18*	Interleukin 18 (interferon-gamma-inducing factor)	1.42	0.0485
*CCR5*	Chemokine (C-C motif) receptor 5	-2.24	0.0117
*FAS*	Fas (TNF receptor superfamily, member 6)	-1.61	0.0049
*ITGB2*	Integrin, beta 2 (complement component 3 receptor 3 and 4 subunit)	-1.31	0.009
*NFKBIA*	Nuclear factor of kappa light polypeptide gene enhancer in B-cells inhibitor, alpha	-1.16	0.0122
*ACTB*	actin, beta	-3.61	0.0031

## Discussion

*Salmonella* poses significant public health and food safety risks, as contaminated poultry products can cause salmonellosis in humans. Controlling *Salmonella* in poultry is crucial to prevent outbreaks and ensure food safety. Vaccination plays a pivotal role in this effort, as it helps prevent *Salmonella* colonization in poultry. In the pursuit of developing effective *Salmonella* subunit vaccines for poultry, researchers have focused on surface-exposed proteins crucial for the bacterium’s survival and infectivity. For example, flagellin (Toyota-Hanatani et al., 2009), *Salmonella* type I fimbriae (De Buck et al., 2005), outer membrane protein OmpC (Ijaz et al., 2025), and type III secretion system (T3SS) proteins (Desin et al., 2011) were explored as potential vaccine candidates, and while they afforded some degree of protection, they did not eliminate colonization of internal organs by *Salmonella*.

In the endeavor to produce subunit vaccines against *Salmonella* that would prevent colonization of internal organs in poultry, this study focused on FepA, an outer membrane protein involved in iron acquisition. Previous studies have shown that iron-regulated TBDTs, crucial for iron acquisition, are vital components influencing bacterial physiology, metabolism, adaptation, and virulence (Dhanani et al., 2015; Tan et al., 2019). Recent transcriptomic analysis of *Salmonella enterica* mutants lacking iron transporters, including *fepA*, *iroN*, and *fhu*, revealed serovar-specific adaptations under iron-deficient conditions (Boamah et al., 2025). Double (Δ*iroN*Δ*fepA*) and triple (Δ*fhu*Δ*iroN*Δ*fepA*) mutants of *S.* Typhimurium and *S.* Enteritidis showed significant changes in gene expression related to virulence, stress response, and energy metabolism. Notably, pathways such as the tricarboxylic acid cycle and electron transport chain were downregulated, while cysteine biosynthesis was upregulated. These shifts suggest that iron limitation triggers broad metabolic reprogramming in *Salmonella*. Importantly, *S.* Enteritidis mutants exhibited enhanced upregulation of flagellar genes compared to *S.* Typhimurium, indicating distinct adaptive strategies between serovars. The study also found that deletion of *fhu* had a limited additional impact compared to the double mutants, suggesting functional redundancy among iron transporters (Boamah et al., 2025). These findings underscore the interconnected roles of iron acquisition, metabolism, and virulence, and further support the rationale for targeting TBDTs like FepA in subunit vaccine development.

Developing vaccines based on membrane-embedded iron-regulated transporters such as FepA poses a significant challenge due to the complexities involved in their recombinant expression, extraction, and purification (Pandey et al., 2016). Their hydrophobic regions tend to aggregate in water, leading to misfolding and reduced stability (Bannwarth and Schulz, 2003). Additionally, membrane proteins often have intricate structures and specific modifications essential for their proper function, making their expression and folding in recombinant hosts error-prone (Kashino, 2003). These challenges hinder the development of effective TBDT-based vaccines.

In the study led by Zarate-Bonilla et al. (2014), employing classical techniques and physicochemical properties, significant antigenic peptides were identified on FepA. These peptides were highlighted as potential candidates for future recombinant or synthetic peptide vaccines. While they shed light on the importance of TBDTs and surface-bound antigenic peptides in vaccine development, it’s important to note that the forecasted peptides were not experimentally synthesized or tested (Zarate-Bonilla et al., 2014). In our research, we utilized the DNAStar software, specifically the Protean 3D package, to predict additional antigenic peptides that were previously undetected. Previous studies have demonstrated the effective use of DNAStar in predicting antigenic peptides (Lon et al., 2020), highlighting its reliability and accuracy. However, the accuracy and suitability of these predictions as vaccine candidates can only be confirmed through subsequent production and immunogenicity studies.

To simultaneously present these predicted antigenic peptides to the immune system, we chose to fuse them in tandem to the N-terminus of BLS, a nanoparticle that enhances the overall presentation of the vaccine candidate to the immune system (Cristófalo et al., 2025). In a study investigating the immunogenic properties of the *Taenia crassiceps* protective peptide GK-1, mice that were orally immunized with GK-1 alone displayed a protection rate of 64.7%. This rate notably increased to 91.8% when GK-1 was co-administered with BLS as an adjuvant and reached an even higher level of 96% when GK-1 was genetically fused to BLS (Fragoso et al., 2011). In another study conducted by Alfano et al. (2015), the inner core domain (VP8d) of the VP8 spike protein from bovine rotavirus was fused to the N-terminus of BLS, generating a fusion protein known as BLSVP8d. This fusion protein was effectively produced in tobacco chloroplasts, and soluble protein extracts from both freshly harvested and freeze-dried leaves triggered the production of specific neutralizing IgY antibodies in a laying hen model (Alfano et al., 2015). These research studies highlight the capability of BLS as a robust platform for developing highly immunogenic subunit vaccines.

The immune condition of the birds used in our study was determined by quantifying the total antibody titers of IgY, IgA, and IgM. IgY, like IgG in mammals, constitutes the predominant immunoglobulin in the birds’ sera. IgY plays a pivotal role in neutralizing toxins, combating pathogens, and interfering with diverse immune processes, making it effective against infections such as *Salmonella* infection in broilers (Gadde et al., 2015). While IgY contributes to systemic immunity, IgA plays a crucial role in mucosal immunity, serving as the first line of defense against pathogens at mucosal surfaces, such as the gastrointestinal and respiratory tracts. IgA achieves this by neutralizing pathogens and preventing their adherence to and penetration of mucosal surfaces​ (Alfano et al., 2015). The consistently elevated levels of general immunoglobulins including IgY, IgM, and IgA in our study indicated the robust immune condition of the trial birds.

Our designed fusion construct, FR-BLS, was expressed at high levels (0.22 mg/g of fresh plant weight) in the cytosol of *N. benthamiana* and its immunogenicity in chickens was tested by quantifying FepA-specific antibodies, ensuring they targeted the FepA antigenic peptide rather than BLS (which functioned as a carrier and adjuvant to enhance immune responses). Our analysis revealed that birds treated with FR-BLS demonstrated notably higher production of FepA-specific IgY antibodies at 28 days old. The emergence of antibodies targeting *Salmonella* within the control groups by day 35 poses a question regarding its origin. One possibility is that the birds may have encountered *Salmonella* during the experiment, leading to this antibody response. To delve deeper into this phenomenon, further investigation and analysis are warranted. In general, a robust IgY reaction was noted, while there was no discernible IgA or IgM response. This outcome aligns with expectations, given that IgA and IgM typically exhibit lower reactivity to injected vaccines. Studies have shown that injected vaccines often induce a stronger IgG response compared to IgA and IgM. For instance, research on COVID-19 vaccines demonstrated that while IgG levels remained high and stable after vaccination, IgA levels decreased significantly faster, and IgM responses were lower in magnitude​ (Wisnewski et al., 2021; Dobaño et al., 2022). This trend is consistent with the known half-lives of these immunoglobulins and their typical behavior in response to injected vaccines (Wisnewski et al., 2021).

Metal ions are essential cofactors of biologic processes, including oxidative phosphorylation, gene regulation, and free-radical homeostasis. The *SLC11A1* gene encodes a divalent metal transporter and participates in cellular iron absorption (Hubert and Hentze, 2002). The upregulation of this gene in FR-BLS-vaccinated birds could be explained by the induction of iron transport process in bursa.

Chemokines are a superfamily of small and soluble proteins whose genes are arranged in clusters. They interact with G-protein-coupled receptor family members, called CC motif chemokine receptor (CCR)1 to CCR5 and are involved in trafficking of lymphoid cells and play a role in host defense mechanisms by modulating the inflammatory response during viral or parasitic infections (Rot and Von Andrian, 2004). We could speculate that the downregulation of this gene in BLS-vaccinated birds could result in decreasing inflammation in the bursa as gene array analysis, chemokine (receptor 5 (CCR5) was found to be upregulated in hepatic cells a murine hepatitis virus-induced model (Liu et al., 2023).

Actins are among the most abundant proteins in eukaryotic cells and involved in functions including cell and intracellular movement as well as muscle contraction. Actins are encoding by various isomeric gene including *ACTC1, 2, 3*, *ACTG1, 2* and *ACTB* (beta-actin). The low-level expression of *ACTB* observed in the present study deserve additional investigation. The deletion of this gene or its loss have been associated with developmental delay, organ malformations, and growth retardation, Baraitser-Winter syndrome, and bleeding disorders (Parker et al., 2020).

While this current study investigated immunizing chickens by injection, *Salmonella* is an enteric pathogen, and mucosal immunity (particularly secretory IgA) plays a crucial role in protecting animals against *Salmonella* infection. Therefore, immunizing chickens with vaccine candidates via routes that stimulate a robust mucosal immune response, such as oral administration, could potentially enhance mucosal antibody production (Lee et al., 2015). Plants are a great choice for oral vaccines for farm animals as they are part of their diet and plant cell walls protect antigens in the gastrointestinal tract (Rice et al., 2005). This approach is cost-effective, requiring no purification, and allows for storage and shipping of dried plant tissue without refrigeration. In Canada, animal vaccines produced in plants and intended for oral administration to animals in dried leaves need to pass regulations related to safety and efficacy and to be registered under the Feed Act and Regulations (Macdonald et al., 2015). As such, veterinary vaccines do not need to go through the same stringent clinical trials as human vaccines, and as a consequence, the path to commercialization of plant-made vaccines is considered to be simpler than that for animals.

Our current study addressed the complexities associated with TBDT expression while leveraging the potential of the outer membrane protein FepA for vaccine development. Our results demonstrated that antibodies were successfully generated against specific FepA loops, rather than the carrier protein BLS. Building on these findings, and considering the leaky nature of TBDTs and their functional redundancy, we plan to design fusion constructs based on various TBDTs involved in iron uptake and immunize chickens with a mixture of these recombinant vaccine candidates. Subsequent *Salmonella* challenge trials in mice and chickens will evaluate the protective efficacy of these candidates against diverse subspecies of *Salmonella enterica.*

## Data Availability

The original contributions presented in the study are included in the article/supplementary material. Further inquiries can be directed to the corresponding authors.
